# Importance of Physical Medicine and Rehabilitation in a Patient With Bilateral Lumbosacral Plexopathy Following the Course of Ladiratuzumab Vedotin for Breast Cancer: A Case Report

**DOI:** 10.7759/cureus.49808

**Published:** 2023-12-01

**Authors:** James Lau, Rayghan Larick, Alyssa Mixon

**Affiliations:** 1 Physical Medicine and Rehabilitation, University of North Carolina at Chapel Hill, Chapel Hill, USA; 2 Physical Medicine and Rehabilitation, Eastern Virginia Medical School, Norfolk, USA; 3 Physical Medicine and Rehabilitation, University of Virginia, Charlottesville, USA

**Keywords:** ladiratuzumab vedotin, physical medicine and rehabilitation, cancer rehabilitation, lumbosacral plexopathy, sgn-liv1a

## Abstract

A 74-year-old female with metastatic triple-negative breast cancer was admitted to the acute care hospital after several ground-level falls and a two-week history of bilateral lower extremity weakness with foot drop, numbness, and tingling. She was on ladiratuzumab vedotin (SGN-LIV1A) and pembrolizumab for four months prior to cancer treatment. Lumbar and sacral imaging studies did not identify neoplastic invasion into the bone or lumbosacral plexus. Electrodiagnostic findings suggested bilateral lumbosacral plexopathy (L3-S1). In the setting of rapid functional decline, medications were reviewed, and SGN-LIV1A was held. On initial evaluation, she required significant assistance with ambulation, transfers, and activities of daily living (ADLs). She remained off SGN-LIV1A and was discharged to acute inpatient rehabilitation. One month following discharge from acute inpatient rehabilitation, she exhibited improvements in right lower extremity strength and foot drop and progressed to modified-independent with ADLs, ambulating with a walker. In a discussion between cancer rehabilitation and oncology with consideration of the timing of presentation, distribution of symptoms, nerve conduction study and electromyography (NCS/EMG) findings, and improvement after SGN-LIV1A discontinuation, the patient was diagnosed with lumbosacral plexopathy from SGN-LIV1A administration. This is the only reported case of lumbosacral plexopathy secondary to SGN-LIV1A and addresses the importance of early consultation with cancer rehabilitation to address sequelae stemming from cancer therapy.

## Introduction

Metastatic triple-negative breast cancer (mTNBC) lacks the expression of the estrogen receptor, progesterone receptor, and human epidermal growth factor receptor 2 (HER2) [1]. Because mTNBC is nonresponsive to endocrine or molecular targeted therapeutics, chemotherapy is currently the first-line treatment [2]. However, mTNBC is generally nonresponsive to chemotherapy, resulting in a poor prognosis with a median overall survival rate of less than two years [3].

Interestingly, 90% of all breast tumors have been shown to express high levels of LIV-1, a transmembrane zinc transporter linked to metastatic progression. Ladiratuzumab vedotin (SGN-LIV1A) is an antibody-drug conjugate (ADC) that utilizes a monoclonal antibody to target LIV-1, resulting in the internalization of conjugated microtubule inhibitor monomethyl auristatin E (MMAE) by mTNBC cells [1,3,4]. Preliminary findings from a recent phase I trial involving mTNBC patients indicate a 32% overall response rate to SGN-LIV1A, prompting further investigation of the ADC as a potential mTNBC therapeutic [3]. Although promising, the adverse effect profile of SGN-LIV1A remains relatively unknown. The drug, SGN-LIV1A, is still under clinical trial and has not yet received United States Food and Drug Administration approval for the treatment of mTNBC. ClinicalTrials.gov identifier: NCT03310957.

We report a unique case of a patient with mTNBC who developed lumbosacral plexopathy while taking SGN-LIV1A. Lumbosacral plexopathy is an injury to the nerves that make up the lumbar and sacral plexus and is characterized by motor and sensory dysfunction of the lower extremities [5]. Lumbosacral plexopathy is a difficult condition to diagnose and treat and can lead to significant functional impairment and morbidity [6]. Early diagnosis and intervention are essential for the preservation of function in patients diagnosed with lumbosacral plexopathy [5]. This case addresses the role of inpatient rehabilitation and importance of early consultation with cancer rehabilitation physicians to address sequelae stemming from cancer therapy.

This case report was previously presented as a poster presentation at the AAPM&R Annual Conference in 2022 [7].

## Case presentation

A 74-year-old female diagnosed with mTNBC presented with a primary right breast tumor 20-50 mm in size with associated metastases to four to nine lymph nodes and the liver (T2N2M1). She was admitted to the acute care hospital for a two-week history of bilateral lower extremity weakness (right greater than left) with associated right foot drop and bilateral distal upper and lower extremity numbness and tingling. She had been taking SGN-LIV1A and pembrolizumab for five cycles over four months per the Seattle Genetics Clinical Protocol [8]. SGN-LIV1A was held throughout cycle 6 due to the aforementioned symptoms. Prior to her admission, the patient was initially treated in an outpatient setting with steroids without improvement and evaluated by outpatient cancer rehabilitation. She was prescribed outpatient physical and occupational therapy but was eventually admitted to an acute care hospital under observation due to rapid functional decline and several ground-level falls. Inpatient Physical Medicine & Rehabilitation (PM&R)/Cancer Rehabilitation were consulted for suspicion of lumbosacral plexopathy secondary to SGN-LIV1A use.

Physical examination showed loss of sensation to light touch in bilateral plantar feet (L>R) and fingertips. Strength testing was significant for 4+/5 right hip flexion, 4+/5 right knee extension, 4/5 right ankle dorsiflexion, and 3/5 right and 4+/5 left extensor hallucis longus extension. On initial physical and occupational therapy evaluations, the patient was below the independent baseline level of function, as she required moderate assistance and a front-wheeled walker (FWW) for ambulation, transfers, and activities of daily living (ADLs).

**Figure 1:**
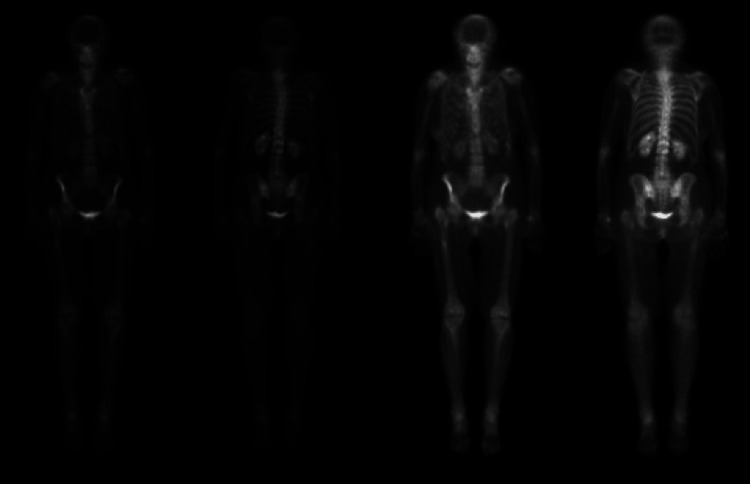
NM whole-body bone scan showed no findings for metastatic bone lesions. NM: nuclear medicine.

**Figure 2:**
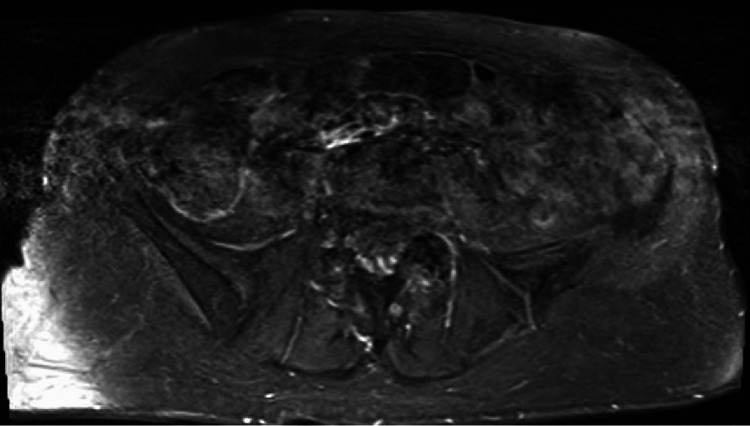
Lumbosacral plexus MRI (T1 fat sat transverse view) unremarkable for any evidence metastatic invasion.

Outpatient electrodiagnostic findings suggested bilateral lumbosacral plexopathy, with dysfunction in the right L4/5 myotome being the most severe (Tables 1, 2). Of note, she was found to have Baastrup’s disease (degenerative changes leading to decreased space between adjacent lumbar spinous processes) around the L4/5 region; however, this did not fully explain her symptoms or exam findings. Brain MRI did not identify abnormalities. Lumbar MRI was significant for degenerative changes in the L3-4 and L4-5 interspinous tissues with moderate spinal canal and bilateral neural foraminal stenosis, consistent with the patient’s Baastrup’s disease. Subsequent nuclear medicine bone scan did not identify any bone metastases (Figure 1). No evidence of neoplastic invasion into the lumbosacral plexus was found on sacrum MRI with and without contrast (Figure 2).

**Table 1:** Nerve conduction study: electrodiagnostic findings suggesting bilateral multilevel lumbosacral radiculopathy and sensorimotor polyneuropathy. NR: no response.

Motor nerve conduction							
Nerve and site	Latency		Amplitude	Segment	Latency difference	Distance	Conduction velocity
Right peroneal							
Ankle	5.3 ms		0.2 mV	Extensor digitorum brevis-ankle	5.3 ms	80 mm	-
Fibula (head)	12.4 ms		0.2 mV	Ankle-fibula (head)	7.1 ms	305 mm	43 m/s
Popliteal fossa	14.5 ms		0.2 mV	Fibula (head)-popliteal fossa	2.1 ms	100 mm	48 m/s
Right tibial							
Ankle	4.3 ms		2.5 mV	Abductor hallucis-ankle	4.3 ms	80 mm	-
Popliteal fossa	15.5 ms		2.0 mV	Ankle-popliteal fossa	11.2 ms	360 mm	32 m/s
Right peroneal							
Fibula (head)	4.2 ms		0.3 mV	Tibialis anterior-fibula (head)	4.2 ms	-	-
Popliteal fossa	6.2 ms		0.2 mV	Fibula (head)-popliteal fossa	2.0 ms	90 mm	45 m/s
Left peroneal							
Ankle	4.8 ms		0.1 mV	Extensor digitorum brevis-ankle	4.8 ms	80 mm	-
Fibula (head)	12.2 ms		0.1 mV	Ankle-fibular (head)	7.4 ms	290 mm	39 m/s
Popliteal fossa	14.0 ms		0.1 mV	Fibula (head)-popliteal fossa	1.8 ms	95 mm	53 m/s
Left tibial							
Ankle	5.2 ms		1.5 mV	Abductor hallucis-ankle	5.2 ms	80 mm	-
Left peroneal							
Fibula (head)	4.8 ms		0.6 mV	Tibialis anterior-fibula (head)	4.8 ms	-	-
Popliteal fossa	6.8 ms		0.5 mV	Fibula (head)-popliteal fossa	2.0 ms	90 mm	45 m/s
Sensory nerve conduction							
Nerve and site	Onset latency	Peak latency	Amplitude	Segment	Latency difference	Distance	Conduction velocity
Right sural							
Lower leg	NR	NR	NR	Ankle-lower leg	NR	120 mm	NR
Right superficial peroneal							
Ankle	NR	NR	NR	Dorsum of foot-ankle	NR	100 mm	NR
Left sural							
Lower leg	3.1 ms	3.9 ms	5 µV	Ankle-lower leg	3.1 ms	120 mm	38 m/s
Left superficial peroneal							
Ankle	NR	NR	NR	Dorsum of foot-ankle	NR	100 mm	NR

**Table 2:** Needle EMG findings suggesting bilateral multilevel lumbosacral radiculopathy and sensorimotor polyneuropathy. EMG: electromyography; MUAPs: motor unit action potentials; Fibs: fibrillations; Fasc: fasciculations; R: right; L: left.

Needle EMG examination									
	Insertional	Spontaneous activity			Volitional MUAPs				
Muscle	Insertional	Fibs	+Wave	Fasc	Duration	Amplitude	Poly	Config	Recruitment
Tibialis anterior R.	Normal	4+	4+	None					
Gastrocnemius (medial head) R.	Normal	2+	2+	None	Normal	Normal	None	Normal	Normal
Vastus medialis R.	Normal	1+	2+	None	Normal	Normal	None	Normal	Normal
Peroneus longus R.	Normal	3+	3+	None	Slight increase	Normal	Many	Normal	Reduced
Tibialis anterior L.	Normal	3+	3+	None	Slight increase	Normal	Many	Normal	Normal
Gastrocnemius (medial head) L.	Normal	2+	2+	None	Normal	Normal	None	Normal	Normal
Vastus medialis L.	Normal	2+	2+	None	Normal	Normal	None	Normal	Normal
Tensor fasciae latae R.	Normal	2+	2+	None	Normal	Normal	None	Normal	Normal
Gluteus maximus R.	Normal	None	1+	None	Slight increase	Normal	Many	Normal	Normal
Lumbar paraspinals R.	Normal	None	None	None	Normal	Normal	None	Normal	Normal
Lumbar paraspinals L.	Normal	None	None	None	Normal	Normal	None	Normal	Normal
Tensor fasciae latae L.	Normal	None	3+	None	Normal	Normal	None	Normal	Normal

Following her acute hospital stay, the patient was transferred to acute inpatient rehabilitation for approximately 14 days. The patient remained off of SGN-LIV1A. Over this time period, the patient participated in physical and occupational therapy for three hours daily, five days per week. Therapy focused on general strengthening, gait training, performing ADLs, durable medical equipment optimization, and family training. Upon completion of acute inpatient rehabilitation, the patient showed notable improvement in function, strength, and ambulation. She was discharged home as modified-independent with FWW. She followed up in the outpatient setting with cancer rehabilitation one month after discharge from acute inpatient rehabilitation with grade 3 motor neuropathy in bilateral lower extremities with altered gait and significant improvement in right lower extremity strength and foot drop with significant improvement in gait function. She continued to follow up with cancer rehabilitation to monitor functional progress. The patient continued working with physical and occupational therapy in the outpatient setting. She remained semi-independent with ADLs and no longer required FWW assistance for ambulation. With careful consideration of the timing of presentation, distribution of symptoms, electrodiagnostic and imaging findings, and improvement after SGN-LIV1A discontinuation, the patient presentation was attributed to lumbosacral plexopathy from SGN-LIV1A administration, and this medication was not resumed.

## Discussion

After a multidisciplinary discussion between cancer rehabilitation and oncology with careful consideration of the timing of presentation, distribution of symptoms, electrodiagnostic and imaging findings, and improvement after SGN-LIV1A discontinuation, the patient's presentation was attributed to lumbosacral plexopathy from SGN-LIV1A administration within the complication of L4/5 radiculopathy secondary to Baastrup’s disease. Despite this pathology having many etiologies, the patient did not have any history of radiation, diabetes, metastases to the lumbosacral plexus, abdominal surgery, or recent trauma prior to her right foot drop [9]. To our knowledge, this is the only report of lumbosacral plexopathy secondary to SGN-LIV1A. 

In addition to highlighting a potential side effect of SGN-LIV1A, our case illustrates the importance of involving both general physiatry and cancer rehabilitation to recognize and address the physical sequelae associated with cancer progression and treatment. Lumbosacral plexopathy is not an uncommon condition, but it remains a very difficult condition to diagnose and treat [5]. The management of lumbosacral plexopathy is dependent on its cause, but the mainstays of treatment include addressing the underlying cause, symptomatic pain management, and rehabilitation [6]. Early identification and intervention are important to reduce morbidity and progression of functional deficits [5]. Cancer rehabilitation specialists are physiatrists with specialized training in the evaluation and treatment of functional disorders caused by cancer and its treatments and can play an important role in early identification and rehabilitative intervention. A systematic review conducted by the World Health Organization identified 69 adult oncology guidelines that recommend either rehabilitative assessment and intervention or referral to rehabilitation services [6]. However, approximately 60% of patients living with or beyond cancer exhibit functional deficits, but only 2-9% of this population have been referred to rehabilitation [9].

The underutilization of rehabilitation services among cancer patients may be attributed in part to a lack of awareness among healthcare providers regarding the benefits of rehabilitation among cancer patients [10]. One study found that only 8% of medical oncologists would recommend admission to inpatient rehabilitation for a patient with functional goals and advanced cancer with six to 12 months of estimated survival [11,12]. For patients with six to 12 months of expected survival, oncologists often recommend hospice and palliative care services rather than rehabilitation, as many perceive the latter to be too physically demanding and time-intensive [11]. Conversely, patients with good prognoses or who have been cured of cancer are rarely referred to rehabilitation because any sustained functional deficits are suspected to be self-limited. However, patients with advanced cancer who underwent palliative care in combination with rehabilitation had improved function, less unmet physical and psychological needs, improved quality of life, and decreased pain compared to those who solely underwent palliative care [13]. Long-term cancer survivors, up to 10 years post-treatment, reported that 38.2% of their unmet needs pertained to physical function [13]. Rehabilitation in cancer patients has been shown to alleviate symptom and caregiver burden, reduce resource needs, and improve quality of life regardless of prognosis, treatment stage, or age [10,11,13].

This case demonstrates the benefits of general inpatient rehabilitation for a patient with metastatic breast cancer as well as the utilization of a multidisciplinary cancer team that includes cancer rehabilitation specialists. This allows for early recognition and evaluation of functional deficits associated with cancer and its treatments, therapy interventions to improve function, and close follow-up to monitor progress.

## Conclusions

With advancements in oncological treatments leading to improved survivorship in the cancer population, there is a need for a multidisciplinary approach that includes cancer rehabilitation experts. Cancer rehabilitation has been shown to alleviate symptom and caregiver burden, reduce resource needs, and improve quality of life regardless of prognosis, treatment stage, or age. The integration of a comprehensive rehabilitative program that addresses cancer- and therapy-associated sequelae into treatment plans early on should be coordinated to prevent worsening complications and functional decline.
